# The miR-1-NOTCH3-Asef Pathway Is Important for Colorectal Tumor Cell Migration

**DOI:** 10.1371/journal.pone.0080609

**Published:** 2013-11-11

**Authors:** Shiori Furukawa, Yoshihiro Kawasaki, Masaya Miyamoto, Masaya Hiyoshi, Joji Kitayama, Tetsu Akiyama

**Affiliations:** 1 Laboratory of Molecular and Genetic Information, Institute of Molecular and Cellular Biosciences, The University of Tokyo, Tokyo, Japan; 2 Department of Surgical Oncology, Graduate School of Medicine, The University of Tokyo, Tokyo, Japan; The University of Hong Kong, China

## Abstract

The tumor suppressor adenomatous polyposis coli (APC) is mutated in sporadic and familial colorectal tumors. APC stimulates the activity of the Cdc42- and Rac1-specific guanine nucleotide exchange factor Asef and promotes the migration and invasion of colorectal tumor cells. Furthermore, Asef is overexpressed in colorectal tumors and is required for colorectal tumorigenesis. It is also known that NOTCH signaling plays critical roles in colorectal tumorigenesis and fate determination of intestinal progenitor cells. Here we show that NOTCH3 up-regulates Asef expression by activating the Asef promoter in colorectal tumor cells. Moreover, we demonstrate that microRNA-1 (miR-1) is down-regulated in colorectal tumors and that miR-1 has the potential to suppress NOTCH3 expression through direct binding to its 3’-UTR region. These results suggest that the miR-1-NOTCH3-Asef pathway is important for colorectal tumor cell migration and may be a promising molecular target for the treatment of colorectal tumors.

## Introduction

Mutations in APC are responsible for sporadic and familial colorectal cancer [1,2]. APC normally induces the degradation of β–catenin and negatively regulates Wnt signaling [3-6]. However, the truncated mutant APCs commonly found in colorectal tumors are defective in this activity. As a result, Wnt signaling is constitutively activated in colorectal tumor cells. APC also stimulates the activity of the guanine-nucleotide exchange factors (GEFs) Asef and Asef2, which are specific for Cdc42 and Rac1 [7-9,12] and enhances their GEF activity, thereby regulating cell morphology, adhesion and migration [7,10,12]. In colorectal cancer cells, mutant APCs aberrantly activate Asef and induce c-Jun amino-terminal kinase (JNK)-mediated transactivation of matrix metalloproteinase (MMP) 9, which is required for tumor invasion [11]. Asef functions downstream of bFGF, VEGF and HGF, and plays a critical role in physiological and tumor angiogenesis [13]. Furthermore, Asef expression is aberrantly enhanced in most human colorectal tumors [11]. Consistent with these findings, we have recently shown that Asef and Asef2 are required for adenoma formation in *Apc* ^Min/+^ mice [11].

 NOTCH genes (NOTCH1~4) encode single-pass, heterodimeric transmembrane receptors that serve as receptors for the Jagged (Jagged1 and Jagged2) and Delta-like (DLL-1, DLL3 and DLL4) ligands. Upon ligand binding, NOTCH receptors are cleaved by γ-secretase, which releases the NOTCH intracellular domain (NICD). NICD translocates into the nucleus and forms a complex with the ubiquitous DNA-binding protein CSL (CBF-1/RBP-Jκ), which then transactivates its target genes, including Hes1, HEY1 and HEY2 [14-17]. NOTCH signaling is a key determinant of intestinal epithelial cell self-renewal and allocation of these cells to specific differentiation lineages [18]. Furthermore, it has recently been demonstrated that NOTCH signaling is important for tumorigenesis in many tissues and organs, including the colon [19-21].

 In the present study, we attempted to clarify the mechanism underlying aberrant Asef up-regulation in colon tumors. Here we show that Asef is up-regulated by NOTCH3 signaling. Furthermore, we demonstrate that microRNA-1 (*miR-1*) is down-regulated in colorectal tumor cells and that miR-1 has the potential to suppress NOTCH3 expression. Moreover, we show that the *miR-1*-NOTCH3-Asef pathway may be important for colorectal tumor cell migration. 

## Results

### NOTCH signaling is important for aberrant overexpression of Asef in colorectal tumor cells

**Figure 1:**
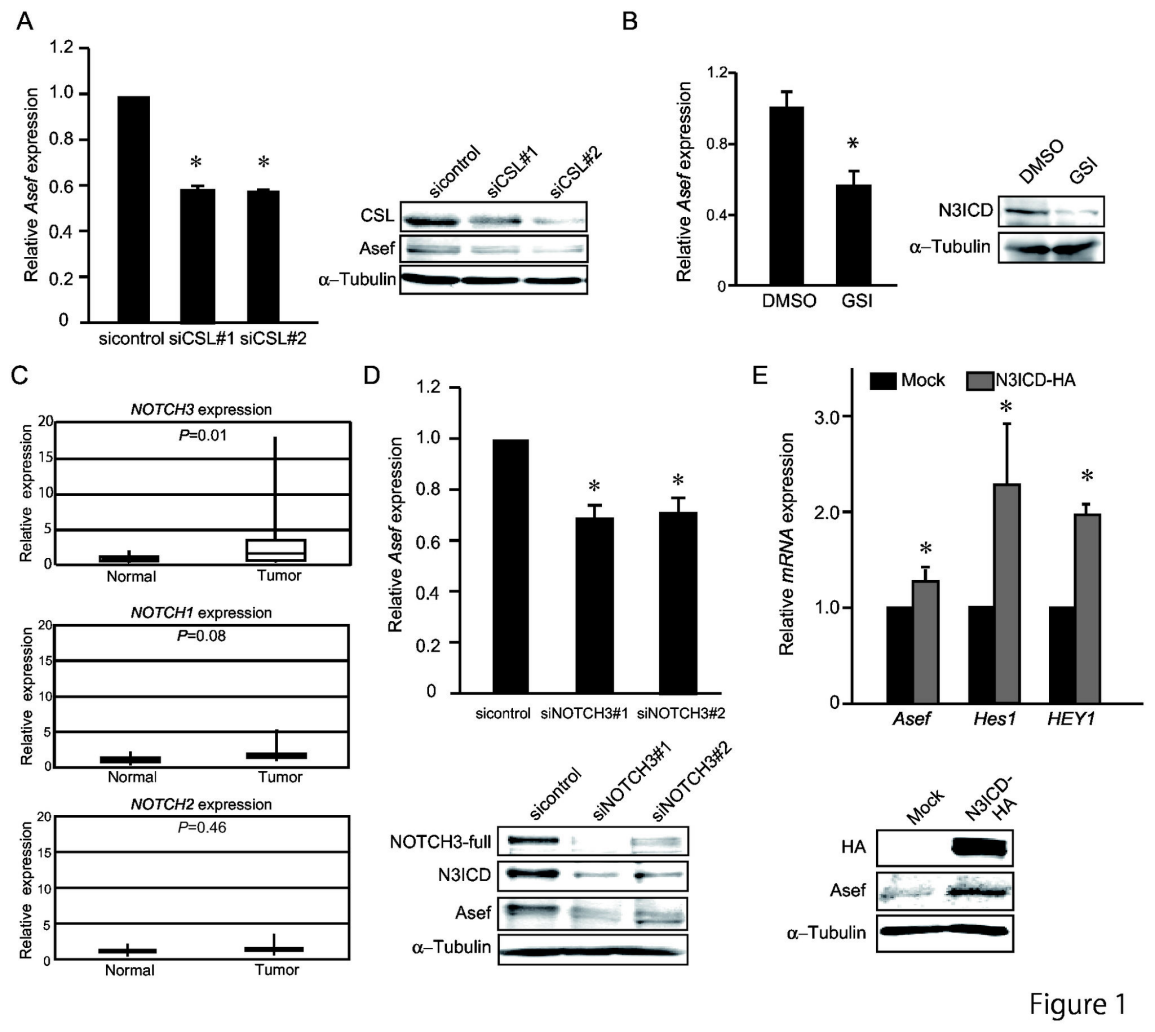
NOTCH3 receptor is up-regulated in colorectal cancerous tissues and regulates *Asef* expression. (A) Knockdown of *CSL* expression by siRNAs downregulates *Asef* expression. DLD-1 cells were transfected with siRNAs targeting *CSL* or control siRNA, respectively and subjected to qRT-PCR of *Asef* (Left) and immunoblotting analysis with the indicated antibodies (Right). α-tubulin was used as a control. (B) DLD-1 cells were treated with the γ-Secretase inhibitor XXI (GSI) or DMSO for 48 h and subjected to qRT-PCR (Left) and immunoblotting analysis (Right). α-tubulin was used as a control. (C) Quantitative analysis of *NOTCH1~3* receptor expression in human colorectal cancers by qRT-PCR. qRT-PCR was performed with 27 pairs of human normal and tumor colon tissues. (D) Knockdown of NOTCH3 expression by siRNAs downregulates Asef expression in DLD-1 cells. The expression of Asef was examined by qRT-PCR (Upper) and immunoblotting analysis with the indicated antibodies (Lower). (E) Effects of N3ICD on *Asef* expression in colorectal cancer cells. DLD-1 cells were transfected with HA-tagged N3ICD and subject to qRT-PCR of the indicated genes (Upper) and immunoblotting analysis with the indicated antibodies (Lower). α-tubulin was used as a control. *p < 0.05.

To identify potential transcription factors that are involved in the aberrant up-regulation of Asef in colon tumors, we searched for transcription factor-binding motifs that are common to the promoter regions of the three *Asef* isoforms: *Asef*-a (Genbank AB042199), -b (Refseq NM_015320) and -c (NM_032995) (Figure S2). Of these, we selected 30 transcription factors (Figure S3) and used siRNA-mediated knockdown to ask whether suppression of any of them affects *Asef* expression in the colorectal tumor cell lines DLD-1, which contain truncated APC. We found that knockdown of CSL caused the most significant decrease in *Asef* mRNA expression, as assayed by Real-time reverse-transcription PCR (qRT-PCR) using the PCR primers common to all isoforms. Immunoblotting analysis with anti-Asef antibody confirmed that knockdown of CSL by siRNA reduced the amount of Asef protein in DLD-1 cells (Figure 1 A). In addition, treatment of cells with the γ–secretase inhibitor XXI (GSI) repressed *Asef* expression (Figure 1 B). 

 The above results suggested that NOTCH signaling regulates Asef expression. We therefore examined the expression levels of NOTCH1~4 in 27 pairs of colon tumors and adjacent normal tissues. qRT-PCR analysis revealed that NOTCH3, but not NOTCH1 or 2 mRNA levels are up-regulated in human colorectal tumor tissues compared to adjacent normal tissues (Figure 1 C). Notch3 mRNA and protein was also overexpressed in intestinal adenomas of *Apc* ^Min/+^ mice (Figure S4). Notch4 was undetectable, as previously reported [22]. We therefore examined whether NOTCH3 is important for Asef expression and found that knockdown of NOTCH3 using siRNA resulted in a decrease in Asef expression (Figure 1 D). Consistent with this result, overexpression of NOTCH3 ICD (N3ICD-HA) resulted in the upregulation of *Asef* mRNA and protein expression (Figure 1 E). These results suggest that NOTCH3 signaling regulates Asef expression in colorectal cancer cells.

### NOTCH3 Regulates Asef Expression by Activating the Asef Promoter

**Figure 2:**
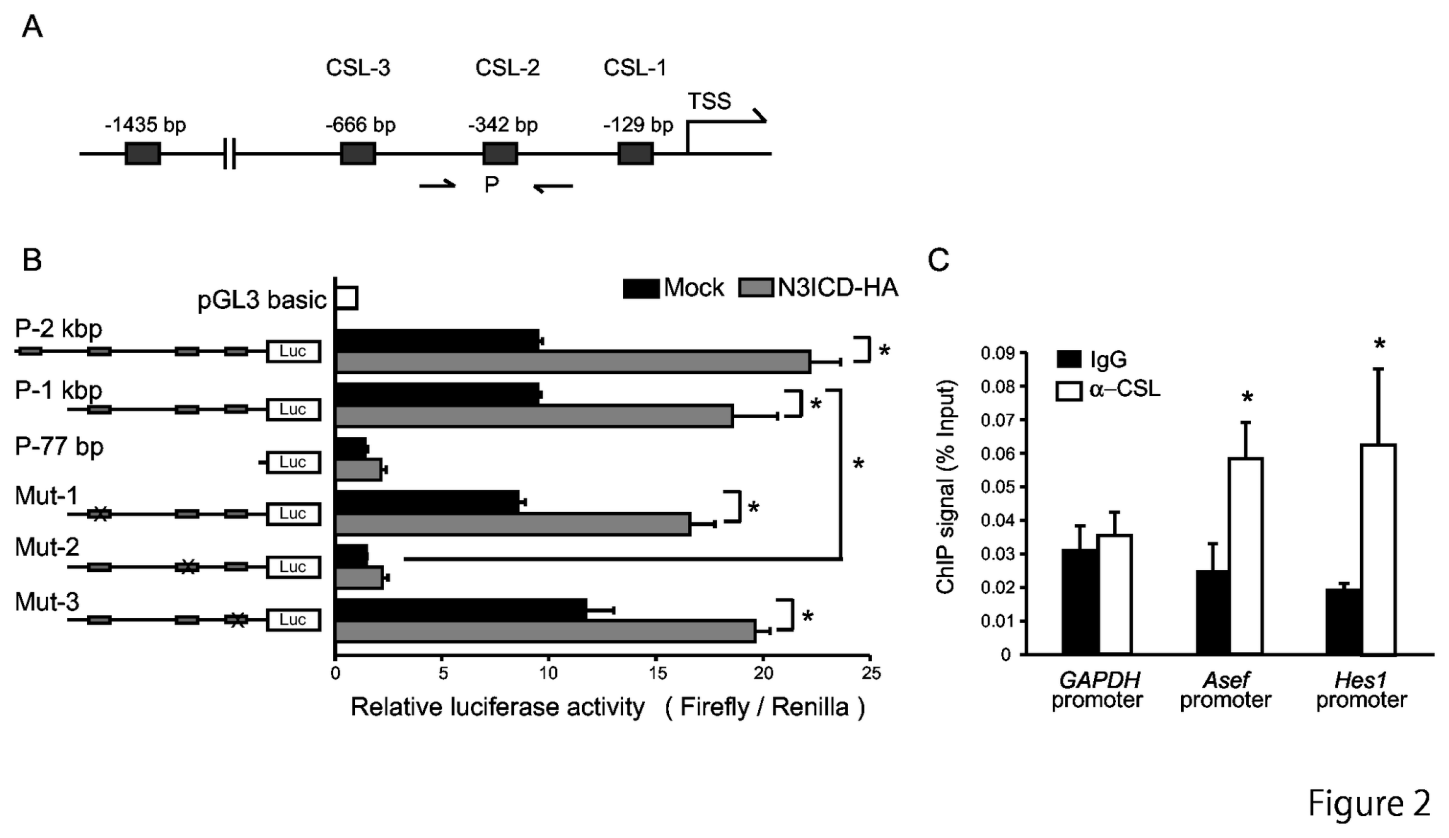
NOTCH3/CSL transactivates the Asef promoter. (A) Schematic representation of the Asef-b promoter region. Predicted CSL-binding sites are indicated by boxes P, the regions corresponding to the primers used for ChIP assays. TSS, Transcription start site. (B) (Left) Schematic diagrams of reporter constructs used for luciferase assays. Fragments of the Asef-b promoter were cloned upstream of the luciferase (Luc) gene. Potential CSL-binding sites are indicated by gray boxes. (Right) Caco-2 cells were transfected with N3ICD or control vector along with reporter constructs containing Asef-b promoter sequences and subjected to luciferase assays. pRL-TK vector was used as an internal control. (C) ChIP assays were performed on DLD-1 cells using anti-CSL antibody or non-specific IgG. The promoter region of *Asef-b* was enhanced in the immunoprecipitates. The promoter regions of *GAPDH* and *Hes1* were used as negative and positive controls, respectively. Results are expressed as the means of the percentage of the input ± SD. *p < 0.05.

We generated a luciferase reporter construct driven by various fragments of the promoter region of *Asef-b*, which is the most highly expressed *Asef* isoform in colorectal tumor cells (Figure 2 A and B; P-2 kbp, P-1 kbp and P-77 bp). These promoter regions contain 4, 3 or no CSL-binding motifs, respectively. When transfected into colorectal tumor Caco-2 cells, the activities of the P-1 kbp and P-2 kbp reporters, but not the P-77 bp reporter, were significantly enhanced by co-expression of N3ICD (Figure 2 B). We also monitored the activities of promoter constructs in which each of the CSL-binding motifs, GGGAA, (CSL-1, -2 and -3 in Figure 2 A) had been replaced with GCTGC (Mut-1, -2 and -3 in Figure 2 B). We found that the activities of Mut-1 and -3 were enhanced by co-expression of N3ICD. In contrast, the activity of Mut-2 was not enhanced by overexpression of N3ICD. These results suggest that the N3ICD-CSL complex up-regulates *Asef* expression by binding to CSL-2 in the *Asef* promoter region.

 To confirm that CSL transactivates *Asef* directly, we performed chromatin immunoprecipitation (ChIP) assays on DLD-1 cell lysates using anti-CSL antibody. We detected CSL binding to a DNA fragment containing CSL-2 (Figure 2 C). The promoter region of *Hes1* was used as a positive control. Taken together, these results suggest that CSL directly upregulates the transcription of Asef by binding to the CSL-binding motif located in its promoter region.

### miR-1 regulates the expression of NOTCH3 in colorectal tumor cells

**Figure 3:**
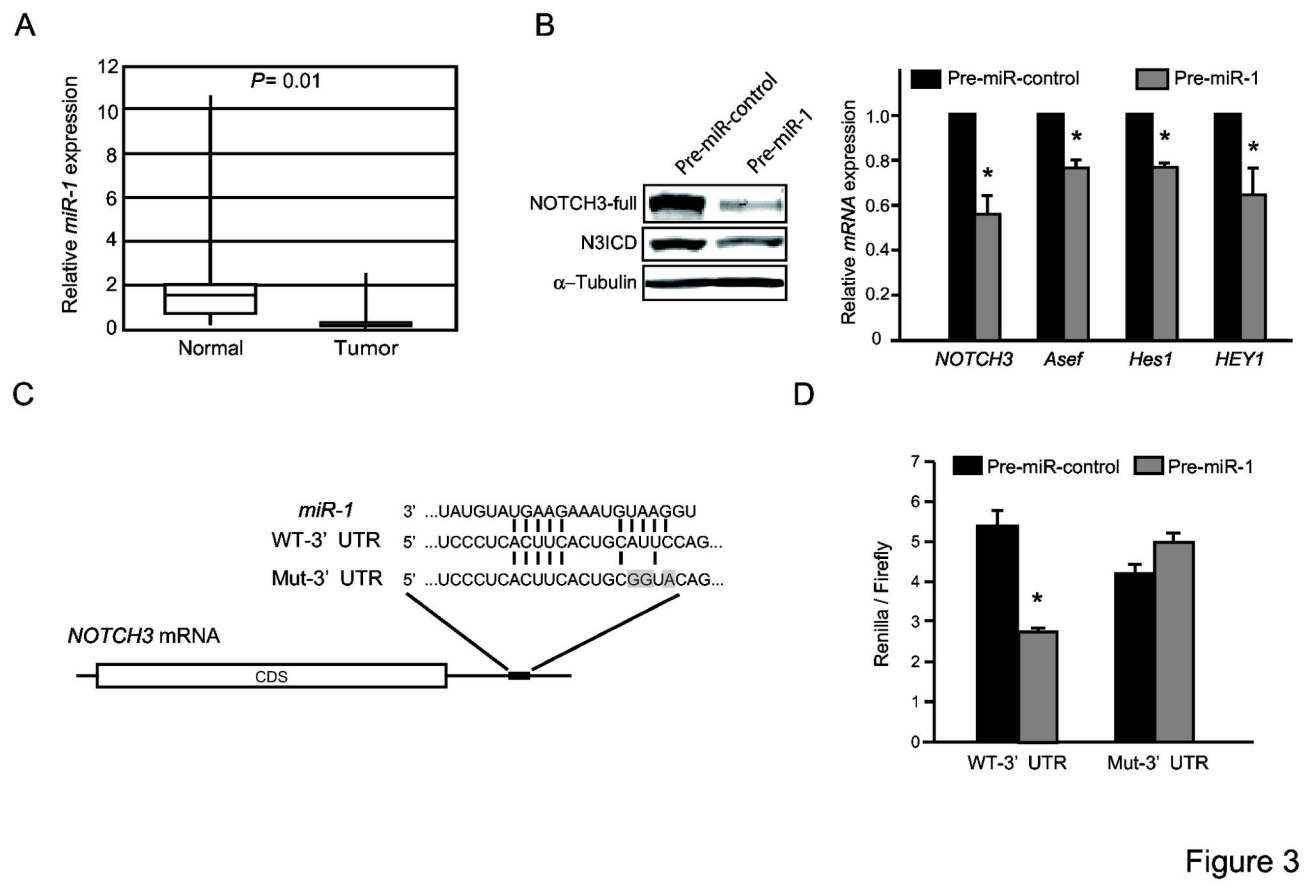
NOTCH3 is a target of *miR-1*. (A) *miR-1* is downregulated in human colorectal cancer tissues. qRT-PCR was performed with 27 pairs of human normal and tumor colon tissues. (B) DLD-1 cells were transfected with Pre-miR-control or -miR-1, and subjected to immunoblotting analysis with the indicated antibodies (Left) and qRT-PCR analysis of the indicated genes (Right). α-tubulin was used as a control. (C) The predicted *miR-1*-binding site in the *NOTCH3* 3’UTR. Sequences of *miR-1* and wild-type (WT-3’UTR) and mutated (Mut-3’UTR) *miR-1*-binding sites are shown. Mutated bases in Mut-3’UTR are marked by gray boxes. Bases in WT- and Mut-3’UTR complementary to those in *miR-1* are indicated by lines. (D) DLD-1 cells were co-transfected with a reporter plasmid and Pre-miR-control or -miR-1, and lysates were subsequently assayed for luciferase. *p < 0.05.

It has been reported that NOTCH expression is regulated by miRNAs [23-25]. To identify miRNAs that directly target NOTCH 3, we performed an in silico search for putative miRNA-binding sites in the 3’-untranslated region (UTR) of the human *NOTCH3* mRNA using TargetScan. We found potential binding sites for *miR-1* and *miR-206* that are conserved among vertebrates. We focused on *miR-1*, as its expression is frequently downregulated in human colorectal cancer tissues. Indeed, as reported previously [26-28], RT-PCR analyses revealed that *miR-1* expression was lower in colorectal tumors than in adjacent normal tissues (Figure 3 A). Overexpression of the *miR-1* oligo, Pre-miR-1, but not the Pre-miR-control in DLD-1 cells led to reductions in NOTCH3 protein and mRNA, as well as N3ICD protein and *Asef* mRNA levels (Figure 3 B). The mRNA levels of NOTCH3 target genes such as *Hes1* and *HEY1* were also decreased.

 To confirm that *miR-1* regulates NOTCH3 expression through binding to the seed sequence in the *NOTCH3* 3’-UTR, we constructed a reporter vector containing the *NOTCH3* 3’-UTR region fused downstream of the luciferase gene (WT-3’UTR;Figure 3 C). As a control, we also generated a luciferase reporter that contains a mutated *miR-1* seed sequence in the *NOTCH3* 3’-UTR region (Mut-3’UTR; Figure 3 C). Transfection of the Pre-miR-1 into DLD-1 cells resulted in decreased luciferase activity from the WT 3’-UTR, but not the Mut 3’-UTR construct (Figure 3 D). 

### The miR-1-NOTCH3-Asef pathway is important for tumor cell migration

**Figure 4:**
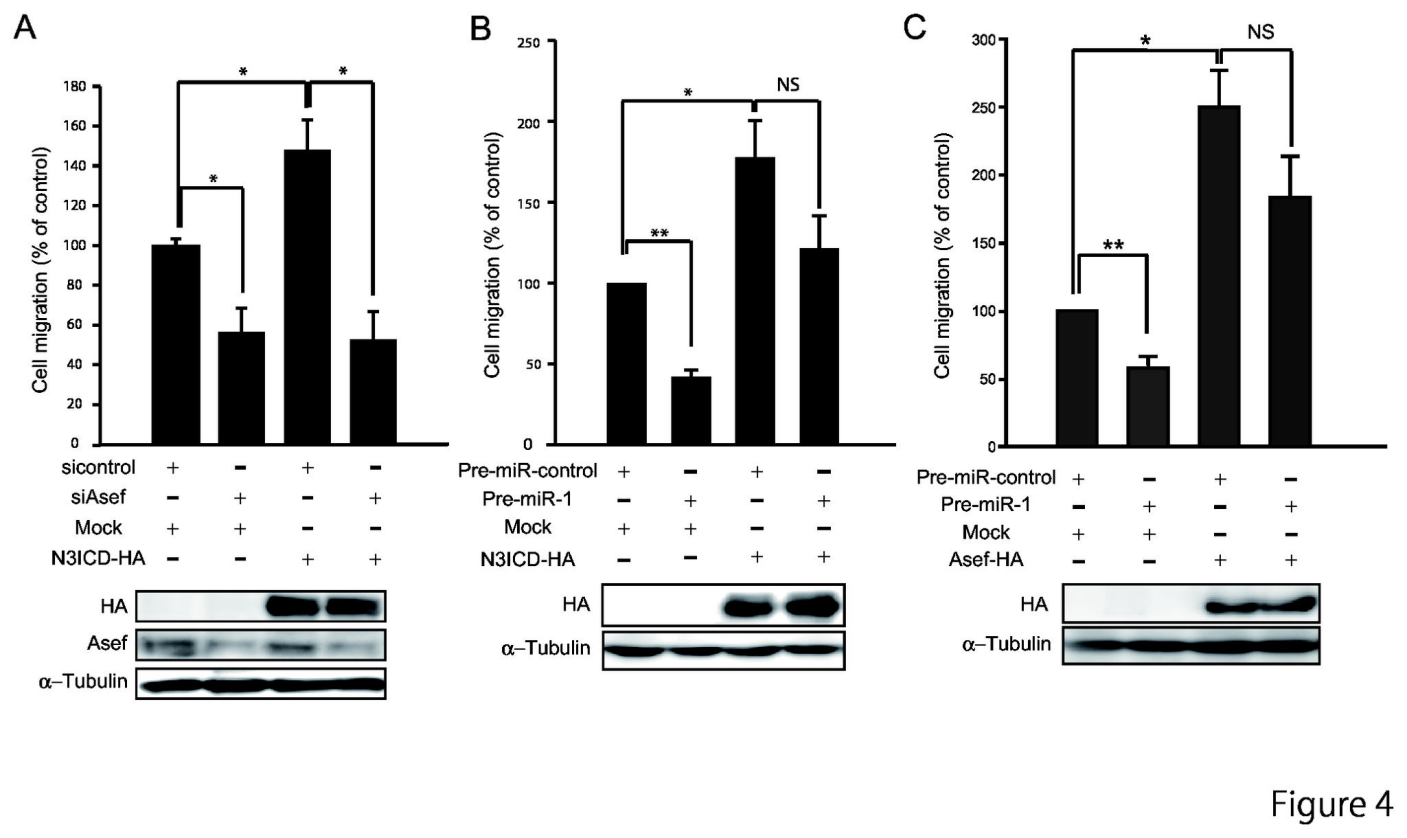
The *miR-1*-NOTCH3-Asef pathway regulates tumor cell migration. (A) Involvement of Asef in N3ICD-induced cell migration. DLD-1 cells were co-transfected with the indicated siRNAs and expression constructs, and subjected to migration assays using Transwell migration chambers (Upper). The expression levels of N3ICD and Asef were determined by immunoblotting with the indicated antibodies (Lower). (B,C) Effects of NOTCH3 and Asef on miR-1-regulated cell migration. DLD-1 cells that had been transfected with Pre-miR-1 were transfected with the indicated expression constructs and subjected to migration assays (Upper). The expression level of N3ICD was determined by immunoblotting with anti-HA antibody (Lower). α-tubulin was used as a control. Results obtained are expressed as the means ±SD of at least three independent experiments. *p < 0.05; **p < 0.01. NS, Not significant.

We next employed transwell migration chambers to examine whether the miR-1-NOTCH3-Asef pathway is important for cell migration. As we reported previously [10], knockdown of Asef by siRNA resulted in a decrease in the migratory activity of DLD-1 cells (Figure 4 A). On the other hand, cells transfected with N3ICD showed significantly increased motility. However, overexpression of N3ICD could not overcome the suppression in migration caused by siRNA knockdown of Asef. These results suggest that NOTCH3 signaling induces cell migration by inducing Asef expression. It has been reported that *miR-1* regulates tumor cell migration [29,30], and we also observed that overexpression of *miR-1* resulted in a marked reduction in tumor cell migration. However, overexpression of either N3ICD or Asef could rescue this *miR-1*-mediated decrease in cell migration (Figure 4 B and C). These results are consistent with the notion that *miR-1* negatively regulates NOTCH3 expression, and thereby Asef expression. Taken together, these results suggest that the *miR-1*-NOTCH3-Asef pathway plays an important role in tumor cell migration. 

### Endothelial DLL4 induces NOTCH3-Asef pathway-mediated cell migration

**Figure 5:**
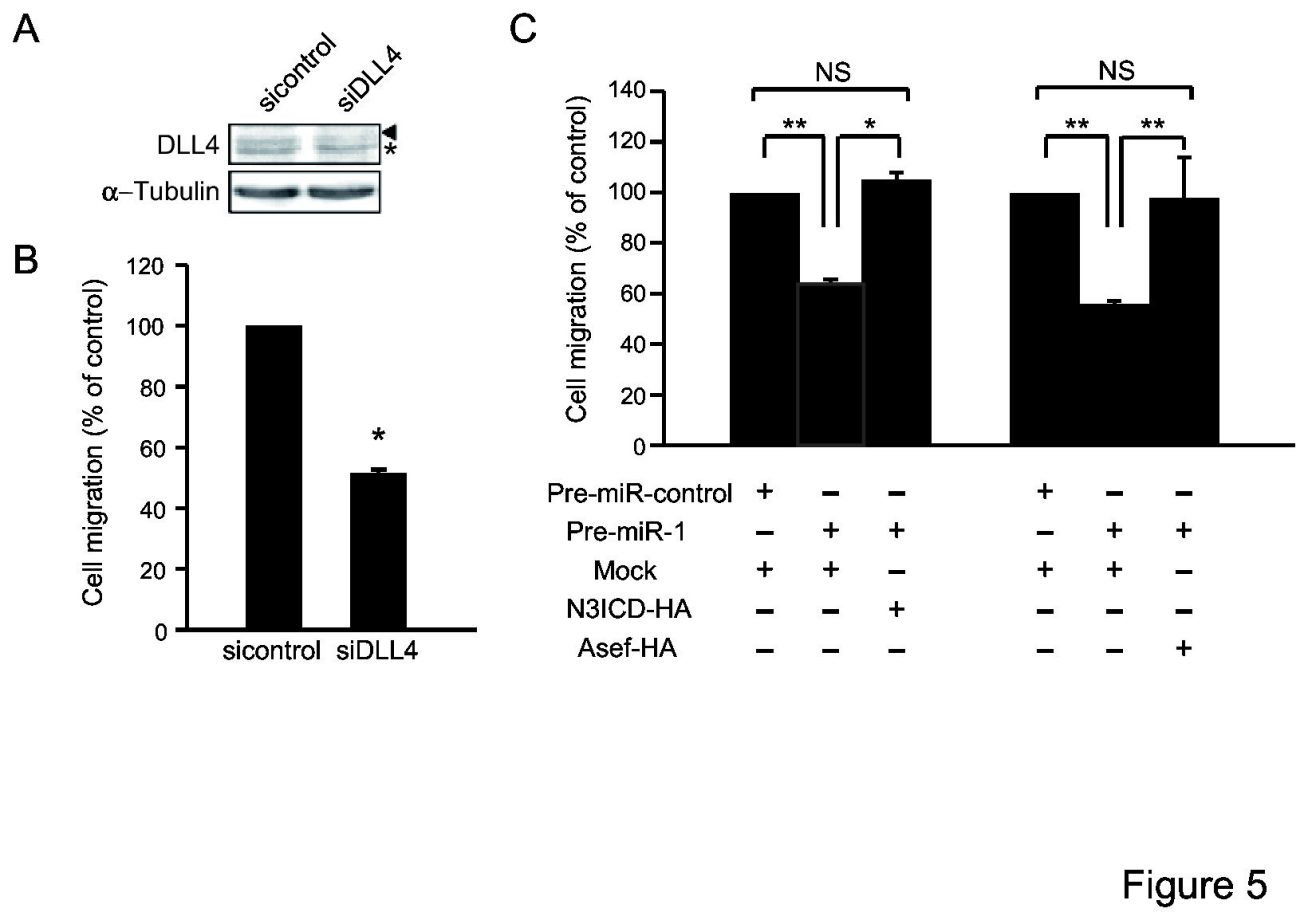
Involvement of endothelial DLL4 in NOTCH3/Asef-mediated cell migration. (A) Suppression of DLL4 expression by siRNA. Lysates prepared from HUVECs transfected with an siRNA targeting DLL4 or control siRNA were analysed by immunoblot with antibodies against DLL4. Antibodies against α-tubulin were used as a control. Arrowhead shows DLL4 band; asterisk indicates a non-specific band. (B) HUVECs transfected with an siRNA targeting DLL4 or control siRNA were grown to confluency on polycarbonate filters in the Transwell chambers. DLD-1 cells were subjected to migration assays. (C) DLD-1 cells that had been transfected with Pre-miR-1 were also transfected with either N3ICD or Asef and assayed for migration activity. Results obtained are expressed as the means ±SD of at least three independent experiments. *p < 0.05; **p < 0.01.

Among the NOTCH ligands, DLL4 has been suggested to be important for the activation of NOTCH3 signaling [31]. Interestingly, it has been reported that DLL4 is highly expressed in blood vessels present in colorectal tumor tissues compared to those in normal colon mucosa [31,32]. We therefore investigated whether expression of DLL4 on vascular endothelial cells stimulates the migration of colorectal tumor cells. We examined the migratory activity of DLD-1 cells using Transwell migration chambers containing a monolayer of human umbilical vein endothelial cells (HUVECs). We found that HUVECs transfected with an siRNA targeting DLL4 were less efficient in stimulating the migration of DLD-1 cells than HUVECs transfected with a control siRNA (Figure 5 A and B). Thus, DLL4 expressed by HUVECs may stimulate migration of DLD-1 cells. We next examined the effect of *miR-1* on DLL4-stimulated migration of DLD-1 cells. We found that DLD-1 cells transfected with Pre-miR-1 showed less migratory activity compared to DLD-1 cells transfected with a Pre-miR-control (Figure 5 C). However, overexpression of either N3ICD or Asef could restore the migratory activity of DLD-1 cells that had been transfected with *miR-1* (Figure 5 C). These results suggest that endothelial DLL4 induces NOTCH3-Asef-mediated colorectal tumor cell migration.

## Discussion

We have previously reported that activation of Asef by truncated mutant APCs present in colorectal tumor cells contributes to their aberrant migratory properties, and that Asef deficiency results in the suppression of intestinal adenoma formation in APC^Min/+^ mice. In the present study, we showed that NOTCH3 signaling induces the expression of Asef in colorectal tumor cells. These findings raise the possibility that mutant APCs and NOTCH3 signaling might cooperate to cause Asef-mediated aberrant colorectal tumor cell migration and adenoma formation. Consistent with this notion, it has been reported that heterozygous deletion of the NOTCH ligand Jagged1 is sufficient to significantly reduce the size of tumors in the APC mutant background [33]. Furthermore, it has also been reported that Wnt/β-catenin is responsible for activating NOTCH signaling in colorectal cancer cells through direct regulation of Jagged1 expression [33]. Thus, we speculate that the NOTCH3-Asef signaling pathway may be a novel therapeutic target for colorectal cancer treatment.

 It has been shown that the *miR-1* family of miRNAs, *miR-1-1, miR-1-2*, and *miR-206*, is frequently down-regulated in many cancers [24-30]. Consistent with previous reports [26-28,34], we found that *miR-1* is down-regulated in colorectal tumors and that *miR-1* has the potential to suppress NOTCH3 expression by binding directly to its 3’-UTR region, which in turn results in a reduction in *Asef* gene expression. Thus, the *miR-1*-NOTCH3-Asef pathway may be critical for colorectal tumor cell migration. Although we found a high correlation (r = 0.88; *p* < 0.001) between the expression ratios (Tumor/Normal) of *NOTCH3* and *Asef*, no significant negative correlation was detected between *miR-1* and *NOTCH3* or *Asef* expression ratios in clinical specimens. 

This result suggests that *NOTCH3* expression is not simply determined by the expression levels of *miR-1*. It remains to be investigated whether the expression levels or functions of factors involved in miR-1-mediated downregulation of NOTCH3 are altered in colon tumors.

 It has also been shown that miR-1 represses the expression of the Notch ligand delta and a downstream target of the Notch pathway, Hes-1 [35,36]. Consistent with previous reports demonstrating that *miR-1* negatively regulates the growth, migration and invasion of cancer cells [29-31,37], we observed a reduction in the migratory activity and growth rate (data not shown) of DLD-1 cells transfected with Pre-miR-1 compared to the Pre-miR-control. Furthermore, we found that overexpression of Asef could rescue the reduced migratory activity, but not the reduced growth rate caused by *miR-1*. This would suggest that the inhibition of growth caused by *miR-1* is mediated by other *miR-1* target genes such as TAGLN2, which is involved in proliferation and apoptosis [30]. 

 It has been reported that *miR-1* expression in many types of cancer cells is down-regulated by epigenetic modifications such as DNA methylation and hypoacetylation of histones. However, we found that treatment with 5-azacytidine, an inhibitor of DNA methyltransferase or trichostatin A (TSA), a histone deacetylase (HDAC) inhibitor had no effect on *miR-1* expression in DLD-1 or Caco-2 cells (data not shown). Thus, the mechanisms by which *miR-1* expression is regulated during tumorigenesis remain to be determined. 

 We found that endothelial DLL4 has the potential to induce colorectal tumor cell migration via NOTCH3 and Asef. It has been reported that DLL4 is highly expressed in tumor-associated endothelial cells in colorectal cancer, suggesting that DLL4 is involved in modulating NOTCH signaling in colorectal cancer cells by heterotypic cell interactions [31]. Furthermore, it has been shown that NOTCH activation by ligands expressed on adjoining blood vessels induces tumor invasion and intravasation in mouse models of colon cancer [38]. The interaction between cancer cells and their microenvironment has a critical role in tumor development and progression. It is therefore tempting to speculate that NOTCH3-Asef signaling may be connected with the invasive behavior of colorectal cancer cells.

## Materials and Methods

### Ethical Statement

The protocol was approved by the Ethics Committee of Tokyo University Hospital and Institute of Molecular and Cellular Biosciences, and written informed consent was obtained from all of the individuals participated in this study. Mouse experiments conformed to the Guide for the Care and Use of Laboratory Animals published by the US National Institutes of Health and was approved by the Ethics Committee of Institute of Molecular and Cellular Biosciences, The University of Tokyo

### Cell Culture and Transfection

DLD-1 and Caco-2 cells were obtained from ATCC and cultured in RPMI640 medium supplemented with 10% FBS and DMEM supplemented with 10% FBS, respectively. Plasmid DNAs, siRNAs and synthesized miRNAs (Pre-miR, Ambion) were transfected into cells using Lipofectamine 2000 or Lipofectamine LTX (Invitrogen). For inhibition of the γ-secretase activity, DLD-1 cells were treated with the γ-secretase inhibitor XXI (25 μM, CALBIOCHEM) for 24 h. 

### Primary human colorectal tissue samples

Human colon cancerous and corresponding non-cancerous tissues were obtained from patients who underwent surgical treatment and who provided informed consent at the Department of Surgical Oncology, the University of Tokyo Hospital. Samples were stored in RNAlater Solution (Ambion) and total RNA was isolated using the mirVana miRNA Isolation Kit (Ambion). cDNA was synthesized using the PrimeScript RT reagent Kit (TaKaRa) (for mRNA detection) or mir-X mRNA First-Strand Synthesis Kit (Clontech) (for miRNA detection). All mRNA data were expressed relative to β-actin and miRNA data were expressed relative to U6 snRNA.

### Plasmid construction and Luciferase assay

N3ICD was amplified by PCR and subcloned into the EcoRI and XhoI sites of the pcDNA3.1 vector with a hemagglutinin (HA) tag. The NOTCH3 cDNA was obtained from Dr. Shigeru Chiba (University of Tsukuba). The promoter regions of Asef were amplified by PCR from the BAC clone RP11-135O12 (Invitrogen) and subcloned into pGL3 Basic. All PCR products were amplified with KOD-Plus- (TOYOBO). Cells were transfected with 500 ng of the firefly luciferase reporter and 50 ng of the pRL-TK vector (internal control). Luciferase activities were measured using the Dual-luciferase reporter assay kit (Promega) with a luminometer (Mithoras LB 940, BERTHOLD). For NOTCH3 3’UTR reporter assays, the NOTCH3 3’UTR was amplified from cDNAs prepared from DLD-1 cells, and PCR products were cloned into the XbaI and NotI sites downstream of the Renilla luciferase in the pRL-TK vector. For co-transfection experiments, pRL-TK (50 ng), pGL3-Basic (10 ng for internal control) and 10 pmol synthetic microRNAs (Pre-miRs) were transfected using Lipofectamine LTX (Invitrogen).

### siRNA-mediated gene silencing

Stealth siRNA duplexes against NOTCH3 and CSL were purchased from Invitrogen. DLD-1 cells were transfected with 20 nM of siRNA using Lipofectamine 2000 (Invitrogen). After siRNA transfection, cells were cultured for 48 h-72 h.

### Immunoblotting analysis

Cells were lysed in RIPA buffer (50 mM Tris-HCl, 150 mM NaCl, 1 mM EDTA, 1% Triton X-100, 0.1% SDS, 0.1% sodium deoxycholate, 1:1000 proteinase inhibitor cocktail). The lysates were subjected to immunoblotting analysis with anti-NOTCH3 (sc-5593, 1:1000; Santa Cruz), anti-CSL (sc-28713, 1:500; Santa Cruz), anti-Asef (NB100-1316, 1:500; NOVUS BIOLOGICALS), anti-DLL4 (ab7280, 1:1000; abcam), anti-HA-tag (3F10, 1:1000; Roche) or anti-α-tubulin (DM1A, 1:500; Calbiochem) antibodies and visualized with BCIP/NBT Color Development Substrate (Promega).

### Real-time PCR

Total RNA was extracted using TRIsure (BIOLINE) and cDNA was synthesized from 1 μg of RNA using the PrimeScript RT reagent Kit (TaKaRa). Real-time PCR was performed in duplicate using the Light Cycler 480 System (Roche) and Light Cycler480 SYBER Green I Master. All mRNA quantification data were normalized to GAPDH (glyceraldehyde-3-phosphate dehydrogenase) expression and compared as differences in ΔΔCt. Primer sequences are listed in Figure S1.

### Chromatin-immunoprecipitation (ChIP) assays

ChIP assays were performed according to the manufacturer’s instructions (Upstate) using anti-CSL antibody (sc-28713, Santa Cruz) or Rabbit IgG (CHEMICON). Precipitated DNA fragments were analysed by real-time PCR using primers directed against a region containing the predicted CSL binding site in the human *Asef* promoter region or the *Hes1* promoter region (positive control). A region in the *GAPDH* promoter region was used as a negative control. Primer sequences are listed in Figure S1.

### Cell migration assay

Cell migration assays were performed using Transwell migration chambers (Costar) as described previously [10]. In brief, after 24 h of transfection, DLD-1 cells (1.0 × 10^5^ cells per well) were resuspended in normal medium and added to the upper compartment of a Transwell chamber and allowed to migrate to the underside of the top chamber for 3 h. Cell migration was determined by counting the cells that migrated to the lower side of the polycarbonate filters. For experiments shown in Figure 5, HUVECs were grown to confluency for 24 h on a polycarbonate filter in Transwell chambers to form a monolayer. DLD-1 cells that had migrated to the lower side of the filter were double stained for DAPI and the endothelial cell marker CD31. CD31-negative cells were counted using fluorescence microscopy.

### Statistical analysis

Statistical analysis was performed using Student`s *t*-test. A P-value <0.05 was considered to be statistically significant.

## Supporting Information

Figure S1 **Primer sequences used for qRT-PCR and ChIP assays.**
(EPS)

Figure S2 **Three isoforms of human Asef.**
(EPS)

Figure S3 **List of 30 transcription factors.**
(EPS)

Figure S4 **Analysis of Notch1~3, Hes1 and Asef expression levels in normal intestinal mucosa and polyps in APC^Min/+^ mice.** (A) Quantitative analysis of *Notch1~3*, *Hes1* and *Asef* expression in mouse intestinal polyps (P) and corresponding normal mucosa (N) by qRT-PCR. mRNA expression was quantified as the percentage relative to *β-actin* mRNA. (n = 4 per group) **p < 0.01. (B) Immunoblotting analysis of normal mucosa and polyps. Lysates from tissues were subjected to immunoblotting with antibodies specific for the indicated proteins. Actin was used as a control.
(EPS)
